# Postglacial recolonization of eastern Blacknose Dace,*Rhinichthys atratulus*(Teleostei: Cyprinidae), through the gateway of New England

**DOI:** 10.1002/ece3.31

**Published:** 2011-11

**Authors:** Michelle L Tipton, Sarah Gignoux-Wolfsohn, Phoebe Stonebraker, Barry Chernoff

**Affiliations:** Biology Department, Wesleyan UniversityMiddletown, CT 06459; College of the Environment, Wesleyan UniversityMiddletown, CT 06459; Department of Earth and Environmental Sciences, Wesleyan UniversityMiddletown, CT 06459

**Keywords:** Connecticut, Blacknose Dace, phylogeography, rapid rates of divergence, recolonization, quarternary

## Abstract

During the last ice age, much of North America far south as 40°N was covered by glaciers (Hewitt 2000). About 20,000 years ago, as the glaciers retreated, the hydrologic landscape changed dramatically creating waterways for fish dispersal. The number of populations responsible for recolonization and the regions from which they recolonized are unknown for many freshwater fishes living in New England and southeastern Canada. The Blacknose Dace,*Rhinichthys atratulus*, is one of the freshwater fish species that recolonized this region. We hypothesize that the earliest deglaciated region, modern-day Connecticut, was recolonized by*R. atratulus*via a single founding event by a single population. In this paper, we test this hypothesis phylogenetically with regard to the major drainage basins within Connecticut. The mitochondrial DNA exhibits low nucleotide diversity, high haplotype diversity, and a dominant haplotype found across the state. A small percentage of individuals in the Housatonic drainage basin, however, share a haplotype with populations in New York drainage basins, a haplotype not found elsewhere in Connecticut's drainage basins. We calculated a range for the rate of divergence for NADH dehydrogenase subunit 2 (*nd2*) and control region (*ctr*) of 4.43–6.76% and 3.84–8.48% per million years (my), respectively. While this range is higher than the commonly accepted rate of 2% for mitochondrial DNA, these results join a growing list of publications finding high rates of divergence for various taxa (Peterson and Masel 2009). The data support the conclusion that Connecticut as a whole was recolonized initially by a single founding event that came from a single refugium. Subsequently, the Housatonic basin alone experienced a secondary recolonization event.

## Introduction

During the Wisconsinan Pleistocene glaciation, approximately 20,000 years BP (years before present), the majority of northern North America above 40°N was covered by glaciers (Dawson 1992; Hewitt 2000; Girard and Angers 2006). This glaciation had many influences on modern vertebrate phylogeography, including reorganizing the distributions of many organisms by forcing them out of their preglacial ranges (Avise and Walker 1998). That organisms are currently found in previously glaciated areas begs the question of how they recolonized these areas.

**Figure 1:**
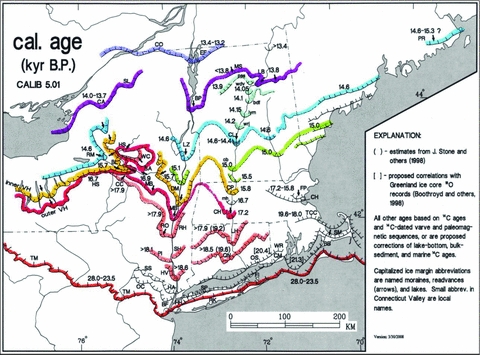
The late Wisconsinan deglaciation varve chronology of the northeastern United States in calibrated (U-Th) ka BP. The numbers along the lines indicate how many thousand years Before Present (kyr BP) the edge of the glacier was there. Arrows indicate ice front positions that are the limits of glacial readvances. Original map from Ridge (2004) and updated on the North American Glacial Varve website.

This study investigates a recolonization of freshwater fish into Connecticut drainage basins that were likely the gateway for fish into the previously glaciated New England region. Connecticut drainage basins were in the first area of New England to become fully deglaciated after the Wisconsinan glaciation (Fig. 1) and were, therefore, the first available for fish recolonization. As deglaciation continued, New York's drainage basins would have been the next available for recolonization by fish waiting in refuge at the glacier's edge. Fishes currently inhabiting waters in New England traveled from a refugium or multiple refugia to the newly formed rivers. There is trace fossil evidence suggesting that the recolonization following deglaciation was rapid, as soon as 75 years after the ice recession (Peteet et al. 1993; Benner et al. 2008, 2009; Knecht et al. 2009).

Varve chronologies provide accurate time scales for the deglaciation of southern New England when used in conjunction with paleomagnetic data and atmospheric ^14^C and U-Th calibration (Stone and Borns 1986; Stone et al. 1998; Ridge 2004; Balco and Schaefer 2006; Balco et al. 2009). Based on these techniques, the edge of the ice margin at Connecticut's coastline is calculated to have existed 20,400 years BP (Fig. 1). The retreat of the glaciers uncovered southern Connecticut at the latest by 18,500 years BP (Boothroyd et al. 1998; Ridge 2004). During early stages of deglaciation (17–15.5 ^14^C thousand years ago [ka]), the meltwater impounded in Long Island Sound forming a temporary freshwater lake, Glacial Lake Connecticut (Stone et al. 1985). When the lake was at lower levels, a drainage system flowed from west to east connecting the mouths of Connecticut's three major drainage basins: the Housatonic, Connecticut, and Thames (Sheet 1, Stone et al. 2005). Eventually, the easternmost edge of the lake impoundment eroded inundating this drainage system with saltwater, thereby isolating the drainage basins.

**Figure 2:**
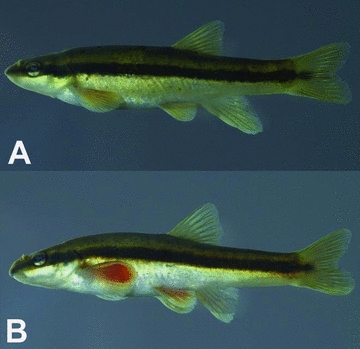
Photograph of eastern Blacknose Dace,*R. atratulus*, from the Coginchaug River (CR; Table A1) in Durham, Connecticut. (A) Female, 62.90 mm SL. (B) Male, 61.35 mm SL. Fish collected by Michelle Tipton on 8 August, 2011 and photographed the same day by Barry Chernoff.

This study examines the genetic diversity of the eastern Blacknose Dace,*Rhinichthys atratulus*(Fig. 2), a small minnow that tends to live in large populations in most of eastern North America, primarily within the Atlantic slope drainage (we distinguish between*R. obtusus*and*R. atratulus*; Nelson et al. 2004).*Rhinichthys atratulus*is an obligate freshwater fish, making it an appropriate candidate for determining recolonization routes as any marine-affected waterways can be eliminated as paths to current distributions. There have been a number of colonization and phylogeographic studies of fish in unglaciated regions of North America (reviewed by Soltis et al. 2006). Little work, however, has been done on fish recolonization within the previously glaciated area of New England. Additionally, there are no phylogeographic studies of*R. atratulus*in the published literature. Our choice to study Blacknose Dace is influenced by this need for more studies on the molecular phylogeography of cyprinids in North America, the most specious group of freshwater fishes. Cyprinids are ubiquitously distributed, and can thus give us insight into the historical, evolutionary, and biogeographical processes of North American rivers (Schmidt et al. 1998; Pfrender et al. 2004). A greater understanding of how postglacial recolonization occurred in this species will provide information on the effect of historic events on modern distributions, gene flow, and conservation in other species (Gilhen and Hebda 2002).

For the purposes of this paper, a recolonization event is the introduction of a species into an area that it had previously inhabited. A founding event is when individuals of a species first enter an area void of that species. The recolonization necessarily consists of multiple or a single founding event(s), which can be followed by other recolonization events. In the case of our study, upon deglaciation the initial recolonization is a founding event; subsequent immigration of populations constitute secondary recolonization, which is not a founding event.

We hypothesize that the recolonization of Connecticut's major drainage basins by Blacknose Dace occurred during a single founding event made up of individuals from one refugium. We test this hypothesis by sequencing the mitochondrial DNA in order to examine the genetic diversity within and between populations, as well as the number and distribution of haplotypes. We also compare Blacknose Dace in Connecticut's drainage basins to individuals in New York drainage basins. We utilize the sociopolitical terms, “Connecticut” and “New York”, because they correlate with the deglaciation patterns–the first and second areas of deglaciation, respectively. Another way to classify these comparisons is by dividing our drainages basins into two groups that correspond to the Hudson River and westward in New York (WH) and those to the east, the three major drainage basins that drain through Connecticut into Long Island Sound (EH). We postulate that EH was the gateway to the recolonization of the freshwaters of New England based on the varve chronology, postglacial hydrologic paths, and our genetic data, which we will further explain below.

The purpose of this study is threefold. First, we investigate whether the recolonization of New England was via a single or multiple recolonization event(s). Second, we examine whether the Blacknose Dace that recolonized Connecticut's major drainage basins came from a single refugium or from multiple refugia. Third, we estimate rates of sequence divergence specific to the mitochondrial gene NADH dehydrogenase subunit 2 (*nd2*) in*R. atratulus*utilizing fossil dated varve chronology as a calibration.

## Methods

### Collection

**Figure 3:**
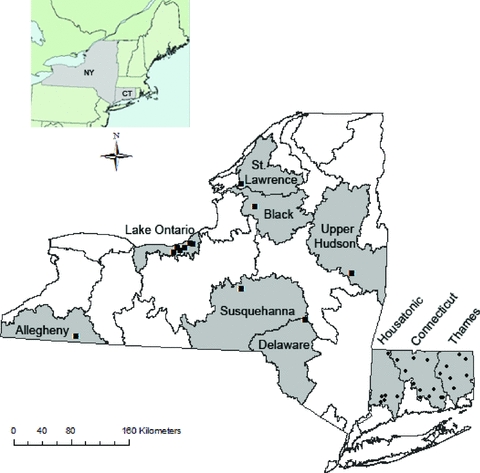
Map of New York and Connecticut with the major drainage basins sampled highlighted in gray and labeled. Sample locations are indicated by black squares in New York and black circles in Connecticut.

Dace were collected from 25 locations across Connecticut (Fig. 3) using a Smith Root backpack electroshocker (Model #: LR-24; scientific collecting permits SC-07014, SC-08022 CT Dept. Env. Protection, Nat. Res. Fish. Div.; IACUC 20110225ChernoffB). Approximately 10 individuals per location were obtained. Fin clips were taken from the caudal fin, and subsequently the fish were released. Scissors were cleaned with 95% ethanol and wiped clean with a Kimwipe. Fin clips were stored in 95% ethanol. An additional 121 samples from various basins across New York State (Fig. 3) were collected in the same way by staff biologists of the New York Department of Environmental Conservation.

### Molecular work

DNA was extracted using a QIAGEN DNeasy Blood and Tissue Kit: QIAGEN Sciences, Maryland, USA. The manufacturer's protocol “Purification of Total DNA from Animal Tissues” utilizing spin columns was followed in order to isolate and purify DNA. The last step of the provided protocol was changed so that 200 µl of buffer was added to the membrane and the samples were incubated at room temperature for 10 min before centrifugation to increase DNA concentration. Final DNA concentration was determined on a Thermo Scientific NanoDrop™ ND-2000 1-position spectrophotometer. This study utilized the*nd2*gene because it is located in the mitochondrial genome, which can be highly variable within species (Avise 2000). The*nd2*gene was amplified by PCR using primers designed by LGL Genetics (Bryan, TX; provided by Phil Harris at the University of Alabama):*nd2*-H: 5′-TGCTTAGGGCTTTGAAGGCTC-3′ and*nd2*-L: 5′-TAAGCTATCGGGCCCATACC-3′. The first half of the mitochondrial control region (*ctr)*was amplified using primers*ctr*-H: 5′-CCRGAAGTAGGAACCAGATG-3′ (Lee et al. 1995) and*ctr*-L: 5′-AACTCTCACCCCTAGCTCCCAAAG-3′ (third nucleotide was changed to a T, Meyer et al. 1994), because it is the more rapidly evolving half (Lee et al. 1995; Broughton and Dowling 1997). We confirmed this for our species by sequencing the entire*ctr*for 12 individuals that had exhibited differences for the first half, and found that the second half was neither more variable nor more parsimony informative (M. L. Tipton, unpubl. ms.). For both regions amplified, the 50-µl reaction volume contained: 0.05 mM of each dNTP, 1.5 mM of MgCl_2_, 0.5 µM of primer, 5 µl of 10× buffer, 1.25 units of Taq (New England Biolabs, Ipswich, Massachusetts, USA), and approximately 250 ng of DNA. Doubly distilled H_2_O was added to reach the final volume. The following reaction conditions were carried out by Applied Biosystem's 2720 (Applied BioSystem, Carlsbad, California, USA) thermal cycler: For*nd2*: an initial denaturation at 94°C for 1 min; followed by 24 cycles of 94°C for 30 sec, 55°C for 1 min, 72°C for 1.5 min; and a final extension period at 72°C for 5 min, lastly holding at 4°C. For*ctr:*an initial denaturation at 94°C for 1 min; followed by 29 cycles of 94°C for 30 sec, 50°C for 1 min, 72°C for 1.5 min; and a final extension period at 72°C for 5 min, lastly holding at 4°C.

Successful amplification of*nd2*and*ctr*was verified by running the samples with 1-µl 6× gel loading dye (NEB) on a 1% agarose gel for 30 min at 100 V. Successful samples, defined by a clear band at ∼1045 base pairs (bp) and ∼450 bp for*nd2*and*ctr*respectively, were sent to Yale University's DNA Analysis Facility for sequencing (New Haven, CT). Forward and reverse sequences were then aligned for each individual using Bioedit–ClustalW Multiple alignment and a consensus sequence was generated.

### Phylogenetic analyses

We used DnaSP v5.10 (Librado and Rozas 2009) to calculate nucleotide and haplotype diversity, π and Hd, respectively (Nei and Kumar 2000). We also performed multiple neutrality tests. Statistics from the following neutrality tests were obtained: Tajima's*D*, Fu and Li'sD*, Fu and Li'sF*, and Fu's*F_s_*.

An Analysis of Molecular Variance (AMOVA) was conducted on the Connecticut population using Arlequin 3.5 (Excoffier and Lischer 2010). The structure used separated populations by sampling site and groups by drainage basin. A statistical parsimony haplotype network with a connection limit set to 95% was generated using TCS (Clement et al. 2000).

### Calculating rates of divergence

The rate of divergence, where*K*is the divergence per million years (my), was calculated using the following equation:*K* = *d*(10^6^)/*t*. K was then multiplied by 100 to create a percentage.*t*represents the years since divergence. A range for*t*was determined based on the varve chronology dates, which establish the earliest possible times that freshwater fish could enter New England river drainages. Two strategies were used in generating the input for this calculation; one in MEGA v4 (Tamura et al. 2007) and the other in BEAST v.1.6.1 (Drummond and Rambaut 2007). This method parallels the usage of biogeographic events as dates in time with which data can be calibrated, as has been done in many studies of the Isthmus of Panama (e.g., Hurt et al. 2009; Miura et al. 2010).

jModelTest 1.1 (Posada 2008) was used to determine the model of sequence evolution using the Akaike Information Criterion (AIC) (Posada and Buckley 2004) for both*nd2*and*ctr*separately. The selected model (Tamura–Nei [TrN]) of sequence evolution was then used to determine*d*using the TrN method in MEGA v4.0 (Tamura and Nei 1993).*d*is the number of base substitutions per site from averaging over all sequence pairs as determined in MEGA, or, it is the root height as determined in BEAST v.1.6.1. The standard error was taken into account for the determination of*d*in MEGA. The range of K was calculated with the adjusted*d*. The upper and lower limits of the root height were used from BEAST to generate appropriate ranges for*d*. In the Bayesian analysis, we generated the xml input file using HKY parameters for BEAST v.1.6.1 in BEAUTi. BEAST v.1.6.1 was run sampling the Markov Chain Monte Carlo (MCMC) every 1000 generations. The output was analyzed in Tracer v1.5, which showed an adequate estimated sample size (>100) and the maximum credibility tree was chosen in TreeStat v1.6.1. Figtree v1.3 was then used to visualize the tree. Root height was chosen as a parameter and calculated by choosing export data in Tracerv1.5. Since we had a mutation rate of one, we transformed this plus root height into the mutation rate across our sequence to get a value of mutations/year/bp, plus the 95% credibility interval for that estimate.

## Results

### Genetic diversity within EH

Of the 1041 nucleotide positions that make up the*nd2*gene, 47 positions were variable (polymorphic) and 18 were parsimony informative. There were a total of 38 haplotypes (GenBank #sJN569201-JN569238; One representative of each haplotype) (Table 1). Hd of the total population was higher than π (Table 2) and the 10^3^ order of magnitude difference between Hd and π was consistent between populations (Table A1). There was one dominant haplotype (haplotype A) that appeared in each river drainage (Table 1). Of the 38 haplotypes, 35 were isolated to a single river drainage and the vast majority of haplotypes were found only in a single population (Table 1).

**Table 1:** The number of individuals per site that represent each haplotype within Connecticut's*R. atratulu*s for*nd2*. The top row indicates the river drainage basin with abbreviations for each of the sites given in the row below (full names in Table A1)

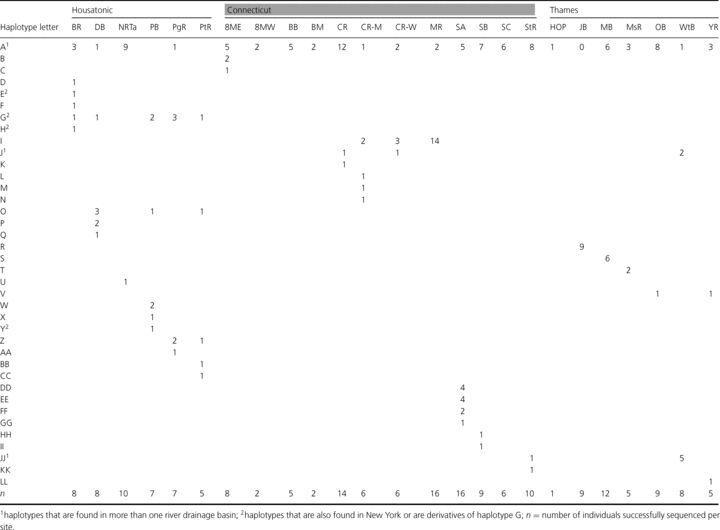

**Table 2:** Population statistics of Connecticut R. atratulus for nd2 by drainage basin. The numbers in parentheses are the standard deviation (SD) or standard error (SE) for that analysis. The TrN model was used to calculate d. The modification of the groups titled “Housatonic Modified” and “All CT Basins with Modified Housatonic,” refers to the removal of 11 samples that are Haplotype G or its derivatives. Similarly, the group titled “All NY Basins Modified” refers to the removal of five samples that were derivatives of CT's haplotype A

Drainage Basin	*n*	No. of Sites	Haplotype diversity Hd (SD)	Nucleotide diversity π (SD)	Average no. of bp differences within group	*d*(SE) Calculated using TrN model
Housatonic	45	6	0.865 (0.036)	0.003 (0.000)	3.125	0.00303 (0.00087)
Housatonic modified	34	6	0.811 (0.060)	0.001 (0.000)	1.437	0.00138 (0.00045)
Connecticut	100	12	0.640 (0.048)	0.001 (0.000)	0.991	0.00095 (0.00034)
Thames	49	7	0.749 (0.050)	0.001 (0.000)	1.026	0.00100 (0.00050)
All CT Basins	194	25	0.755 (0.032)	0.002 (0.000)	1.643	0.00159 (0.00038)
All CT Basins with modified Housatonic	183	25	0.727 (0.035)	0.001 (0.000)	1.141	0.00110 (0.00028)
All NY basins	91	9	0.669 (0.057)	0.002 (0.000)	1.668	0.00162 (0.00045)
All NY basins modified	86	9	0.629 (0.061)	0.001 (0.000)	1.089	0.00105 (0.00033)

For*nd2*, all of the neutrality tests conducted were statistically significant when calculated using the entire set of samples from drainages east of the Hudson River (EH). Tajima's*D*was statistically significant at –2.36045 (*P* < 0.01). Fu and Li'sD* test statistic was –5.90391 (*P* < 0.02), Fu and Li'sF* test statistic was –5.24763 (*P* < 0.02), and Fu's*F*_s_ statistic was –41.048 (*P* < 0.00). The AMOVA results were significant (*P* < 0.001; ±0.000) for all categories when looking within and among drainage basins for all individuals (Table A2). However, when the haplotypes that were derivatives of or matching the Hudson River drainage basin and basins to the west (WH) haplotypes were removed from the Housatonic, the variance within populations was no longer significant (*P* = 0.119; ± 0.011; Table A2).

The average number of nucleotide differences for all basins was 1.643 and nucleotide diversity, π, was 0.002 (Table 2). Haplotype diversity within each site ranged from 0 to 1.00. Nucleotide diversity ranged from 0 to 0.003. Six different populations had both haplotype and nucleotide diversities of zero, indicating that all of the individuals at a site shared the same haplotype. The number of haplotypes at a site ranged from one to seven.

The highest haplotype diversity was found in the Housatonic drainage basin and the lowest in the Connecticut drainage basin (Table 2). Similarly, the Housatonic had the highest nucleotide diversity, while the Connecticut and the Thames drainage basins had appreciably lower nucleotide diversities (Table 2). The average number of nucleotide differences for the Connecticut and Thames drainage basins were similar. In the Housatonic, however, the average nucleotide difference and calculated*d*were triple that of the other two drainage basins (Table 2).

We were able to sequence a 451-bp long portion of the first half of the*ctr*. Only 11 of the 451 nucleotide positions were variable (polymorphic). Six sites were parsimony informative. Of the 199 individuals successfully sequenced for*ctr*, only 12 haplotypes were found (GenBank #sJN569263-JN569274; One representative of each haplotype). Hd for EH was 0.369 and π was 0.001. There was a single haplotype that dominated the population, found for 79% of the individuals.

For*ctr*, two of the four neutrality tests were statistically significant. Tajima's*D*was not statistically significant at –1.66371 (0.10 >*P*> 0.05). Fu and Li'sD* test statistic was not statistically significant at –2.29375 (0.10 >*P*> 0.05). Fu and Li'sF* test statistic was statistically significant at –2.47015 (*P* < 0.05). Fu's*F*_s_ statistic was significant and negative with –9.211 (*P* = 0.000). The AMOVA results were significant (*P* < 0.001; ± 0.000) for all categories within and among drainage basins for all individuals (Table A3). The genetic diversity for*ctr*did not change when the individuals identified for*nd2*as WH haplotypes were removed.

### Comparison of genetic diversity of (EH) to (WH)

**Figure 4:**
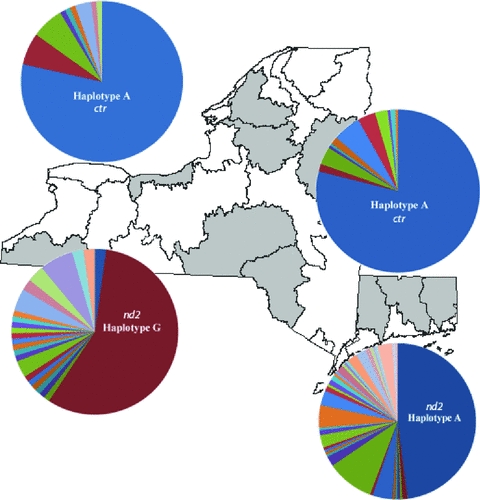
Frequency of each*R. atratulus*haplotype in the respective state's population. The sample size of each population for*nd2*is*n* = 194 for Connecticut and*n* = 91 for New York. The pie charts display 38 and 24 haplotypes. The two dominant haplotypes for each state are labeled as Haplotype A and Haplotype G. For*ctr n* = 199 and displays 12 haplotypes within Connecticut and*n* = 93 and displays nine haplotypes for New York.

Similar to EH, WH had a dominant haplotype, listed in Table 1 as haplotype G for*nd2*(Fig. 4). Haplotype G was found in each sampled WH drainage basin with the exception of the Allegheny where only*R. obtusus*, the western Blacknose Dace, was found. EH's dominant Haplotype A was not found in WH, but WH's dominant haplotype G was found in EH. Haplotypes characteristic of WH (haplotype G and its derivatives, Table 1; Genbank #s JN569239-JN569262; One representative per haplotype for all WH samples) were restricted to the Housatonic drainage basin, and they were found at every Housatonic site except one: the Naugatuck river tributary (NRTa; Table A1). The Housatonic drainage basin is the western major drainage basin and, therefore, closest to New York, but still east of the Hudson River (Fig. 3). Similar to EH's drainage basins, haplotype isolation by drainage occurred in WH.

For*ctr*, the dominant haplotype for WH and EH were one and the same: haplotype A. Of the 93 fish sequenced for*ctr*in NY drainage basins, 73 were haplotype A. This 78.5% dominance of haplotype A in WH's population is very similar to EH's 79% dominance. The combination of the two populations raised the number of haplotypes from 12 to 18 (Genbank #sJN569275-JN569280; One representative per haplotype), increasing the Hd to 0.3725.

**Figure 5:**
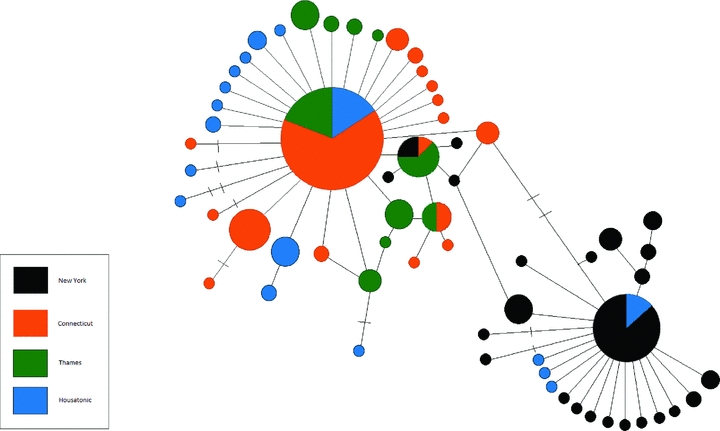
Ninety-five percent statistical parsimony haplotype network (created in TCS) showing the relationship of New York and Connecticut haplotypes for*nd2, R. atratulus*only. Each circle represents a single haplotype (defined by one nucleotide difference). Every hash mark represents one additional nucleotide difference. Each haplotype is color coded according to how many individuals displaying that haplotype were found in the Housatonic, Connecticut, or Thames drainage basins or in the state of New York. The size of the circle corresponds to the frequency of individuals found with that haplotype. The large circle on the left is Connecticut's haplotype A and the largest circle of the cluster on the right is New York's haplotype G.

The haplotype network for*nd2*with both WH and EH samples show that WH's dominant haplotype G connects through multiple pathways to EH's dominant haplotype A (Fig. 5). Only three of the 40 individuals that stem from haplotype G are from EH sites (Fig. 5). The haplotype network also shows the relatively even distribution of haplotypes among the EH as well as the overall low nucleotide and high haplotype diversities. Another haplotype network (not shown) including the outgroups,*R. obtusus*and*R. cataractae*, reveals that they connect to EH's dominant haplotype A prior to the connection with WH's dominant haplotype G. This rooted result shows EH's haplotype A to be basal to WH's haplotype G.

### Calculation of rate of divergence

**Figure A1:**
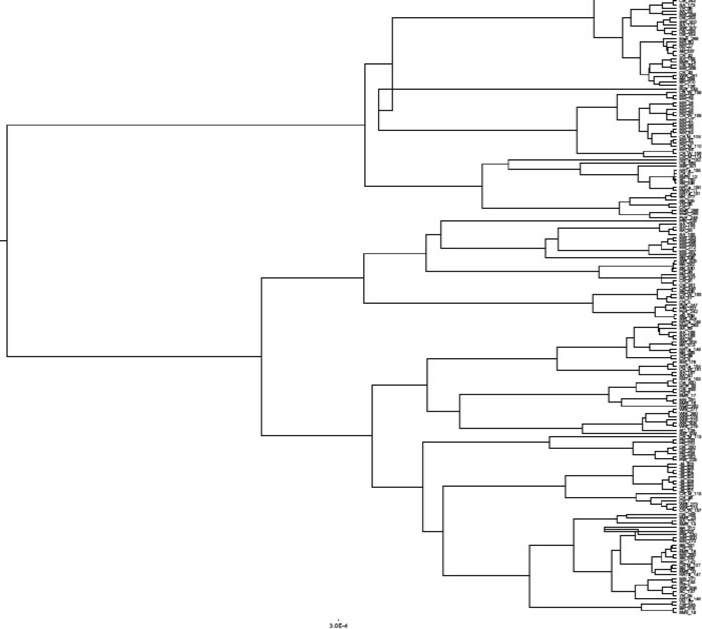
Maximum credibility tree from BEAST output of all CT samples chosen in TreeStat v1.6.1. Tree was visualized in Figtree v1.3.

jModelTest determined, using the AIC, that the appropriate model of sequence evolution for*nd2*and*ctr*(run separately) in the EH population was TrN model. In order to assess the mutation rate of the founding population of EH, samples that were haplotypes E, G, H, and AA were omitted because they were genetically distinct and thus deemed not part of the founding population in EH. With MEGA4, we calculated for*nd2*that*d* = 0.0011 (SE = 0.00028) (Table 2) for the EH samples when excluding the 11 samples that contained WH's haplotype G or its derivatives as indicated above. Similarly,*ctr*'s*d* = 0.0012 (SE = 0.00051) (the same 11 samples were excluded for consistency). Given the earliest approximate time (*t*) that the dace could have recolonized the area, 18,500–20,400 years BP, we calculate the rate of divergence (*K*) as a range of 4.43–6.76% per my for*nd2*. Similarly in MEGA, we calculated*K*for*ctr*to be 3.84–8.48% per my. The root height calculated in Tracer v.1.5 from the BEAST v.1.6 file at 95% HPD was 0.00265 with a standard deviation of 0.00001. The generated Bayesian tree with 95% credibility intervals is in Appendix A (Fig. A1). The 95% HPD lower limit was 0.00142 and the 95% HPD upper limit was 0.004175. When these upper and lower limits are used for the root height and then used as*d*, we calculate that the range for*K*is 7.68–20.47% per my.

We calculated*d* = 0.00052 for individuals in the Housatonic drainage basin that have*nd2*'s haplotype G or derivatives of haplotype G. Using this value for*d*and the more conservative*K*of 4.43–6.76% per my, we calculate that haplotype G has been in the Housatonic drainage for 4,738–12,206 years. Using the same method (*d* = 0.00105; SE = 0.00033, All NY Basins Modified, Table 2), we calculated that haplotype G has existed in WH twice as long as it has existed in the Housatonic: 16,244–20,294 years. Additionally, we divide the number of nucleotide differences between haplotype A and G by the length of the sequence to determine that*d* = 0.0038. Given that*K* = 4.43–6.76% per my, we then use these values to determine the length of time (*t*) since divergence of haplotypes A and G—between 71,260 and 86,690 years BP. Since this amount of time is a conservative estimate, we also calculated*t*using the BEAST's root height, resulting in a range of 49,145–59,787 years BP. In either case, the time range places their divergence during the Wisconsinan glaciation.

## Discussion

In this paper, we investigated the number of refugia, the number of recolonization events, and rates of divergence in postglacial eastern Blacknose Dace populations in Connecticut's major drainage basins, the gateway to New England. We hypothesized that the most parsimonious scenario of recolonization of Connecticut's major drainage basins (EH) by the Blacknose Dace is a single founding event from a single refugium. We identified dominant haplotypes, haplotype A and G. According to our calculated ranges, using both MEGA and BEAST, haplotypes A and G's most recent common ancestor occurred between 49,145–86,690 years BP, which predates the deglaciation time period. This supports the idea that these two haplotypes would have arisen in separate refugia. The difference in haplotype and nucleotide diversities between the Housatonic and the other two drainage basins implies that EH as a whole contains fish from multiple refugia. That the elevated diversities are isolated within the Housatonic drainage basin and are driven by 11 individuals that match or are derivatives of haplotypes found in WH, however, indicates that these individuals were from a second refugium not involved in the initial recolonization event of EH. Assuming that these elevated diversities are due to a subsequent recolonization event, omitting the Housatonic samples from some of our analyses allows for characterization of the population involved in the founding event only. This modified population exhibits a dominant haplotype and low nucleotide diversity across EH indicating that it experienced recolonization by a single founding event involving a single refugium. The data fail to refute the null hypothesis and the details will be discussed in more detail below.

### A founding event and subsequent recolonization event

When looking at the more parsimony informative*nd2*gene, the Housatonic drainage basin exhibited three times higher haplotype and nucleotide diversities when compared to EH's other two drainage basins (Table 2). This indicates that there was one founding event for all of EH followed by a subsequent recolonization isolated to the Housatonic drainage basin. In order to investigate this anomaly of high diversity, we incorporated populations from WH into our analyses. We found that the Housatonic and WH had one haplotype in common: WH's dominant haplotype G. Haplotype G is not found in EH besides the Housatonic. The isolation of haplotype G to Connecticut's western most drainage basin, as well as its dominance in WH, implies that it was introduced into Connecticut's Housatonic drainage basin during a subsequent, more recent recolonization event. We calculated that haplotype G has existed in WH (16,244–20,294 years) twice as long as in EH (4,738–12,206 years), further indicating that it was introduced into EH much later than the founding event.

The neutrality tests further support a single founding event. Since Fu's*F*_s_ is particularly sensitive to recent genetic expansions (Fu 1997), our data with a highly significant*F*_s_ for both genes indicate that the EH population was founded by individuals from a low-diversity founding population or bottleneck event, which they have subsequently expanded from. A single founding event was logistically possible due to the existence of a temporary freshwater river in Long Island Sound that connected the major drainage basins in EH during deglaciation (Stone et al. 2005).

### Founding event from single refugium

We removed haplotype G and its derivatives from the Housatonic drainage basin population, lowering nucleotide and haplotype diversity to that of the other drainage basins. By excluding haplotype G and its derivatives from the analysis of EH populations, we are able to determine the number of refugia involved in the founding event. For*nd2*, the single dominant haplotype A is found in similar frequency across EH. There are two other haplotypes that are found across two of the three major drainage basins, but are in a much lower frequency. We also know that haplotype A is basal to haplotype G. Thus, we infer that haplotype A was present in the founding population at a high frequency, as well as the other two haplotypes, but at a lower frequency. The remainder of the haplotypes that are found in EH arose from haplotype A after recolonization due to isolation by distance (Wright 1943; Avise 2000). This is based on the facts that: one, they are isolated by basin and often by site (Table 1); two, the haplotype network shows that they all radiate off of haplotype A, often differing by only a single nucleotide and exhibit low nucleotide diversity (Fig. 5). The AMOVAs for both genes determined that the drainage basins are significantly different (*P* < 0.001;*P* < 0.05;*nd2*and*ctr*, respectively), corroborating the isolation of the 35 derivative haplotypes within drainage basins (Table 1). The AMOVA results and haplotype isolation by drainage indicate that these derivative haplotypes had to have arisen following their establishment in each basin. This distribution of haplotypes supports our parsimony hypothesis of a single founding event.

The genetic diversity of Blacknose Dace in EH consists of a dominant haplotype found in every drainage basin and nearly every site, with regard to both genes (Table 1 and Table A3). This prevalence of a dominant haplotype signals a single ancestral source (Gugerli et al. 2009). Additionally, low nucleotide diversity and high haplotype diversity indicates a single refugium (Peters et al. 2005). This ratio results in shallow mtDNA lineages lacking distinct clades in a population. In contrast, high nucleotide diversity and high haplotype diversity would cause deeply divergent lineages with multiple distinct clades, indicating multiple refugia (Peters et al. 2005). Our haplotype network lacked distinct clades (Fig. 5), supporting a single refugium hypothesis (Templeton 1998).

The neutrality tests further support the hypothesis that recolonization came from a single refugium. The significant negative values for Fu and Li'sD* andF*, as well as Tajima's*D*indicate an excess of rare alleles with few intermediate haplotypes. This suggests that Blacknose Dace populations in EH have undergone a recent expansion (Venkatesan et al. 2007). The low genetic diversity and single dominant haplotype of the presumptive founding population point to recolonization of Blacknose Dace into EH from a single refugium. Previous studies have shown postglacial dispersal to have originated from multiple refugia (e.g., Rowe et al. 2004; Steele and Storfer 2006; Hoarau et al. 2007; Aldenhoven et al. 2010; Tang et al. 2010), as well as single refugium scenarios (e.g., Gaudeul 2006; Gugerli et al. 2009; Moncrief et al. 2010). A low-diversity founding population is most parsimoniously derived from a single refugium, and that there was a simultaneous recolonization from multiple refugia containing the same genetic signature and low genetic diversity seems highly unlikely.

### Rates of divergence

Here, we utilize the varve chronology dates and the following rationale to derive the calculations of rates of divergence. There is precedent in the work done in Central America to use biogeographic features as a calibration point when fossils for the species are lacking. The closing of the Isthmus of Panama has been used in many studies as a calibration point for determining rates of divergence (e.g., Hurt et al. 2009; Miura et al. 2010), but no studies to this date have used varve chronology that is established with paleontological and geological data. Benner et al. (2009) suggest that varve chronology and trace fossils may be used as a calibration for methods of determining rates of divergence and the migration pathways for some modern coldwater fish species. Due to the small number of freshwater fish skeletons and trace fossils found in the Northeast region, we employed the calibrated and dated varve chronology record. Given that recolonization of EH was likely rapid (Peteet et al. 1993; Benner et al. 2008, 2009; Knecht et al. 2009), we assumed that approximately 20,000 years BP, during the beginning of glacial retreat, marks the earliest possible arrival of primary freshwater fishes to the area (Ridge 2004). The mouths of the rivers were connected 17–15.5 C^14^ ka (Stone et al. 2005). Based on the isolation of haplotypes by basin, the drainage basins being a few million years old and the earliest possible recency of when the fish could have entered Connecticut's rivers, we postulate that all haplotypes isolated with a basin have arisen since deglaciation. This is supported by results from the AMOVAs of both genes that were significant within drainages at the site level, which means that there is significant structure of contemporary genetic isolation due to river barriers.

It is a common mistake for rates of divergence to be given as an absolute value. Because of the amount of error that is inherent in the calculation, this value is better represented as a range (Ho 2007). The calculations presented in this paper utilize the range of earliest possible dates of entry by the fish into EH so as to account for error. Additionally, our calculated range of 4.43–6.76% per my and 3.84–8.48% per my,*nd2*and*ctr*, respectively, differs from conventional rates of divergence for mitochondrial genes. Generally, a standard rate of 2% per my is assumed for all mitochondrial genes across species (Paxinos et al. 2002; Ho 2007). In the sister species of*R. atratulus, R. cataractae*, and*R. obtusus*, Smith and Dowling (2008) used fossils to determine a single-lineage divergence rate of 1.8% per my for*Cyt b*, another mitochondrial gene. Our calculated 4.43–6.76% and 3.84– 8.48% rates, therefore, indicate that*nd2*and the first half of*ctr*mutate at a faster rate than*Cyt b*in this group of fishes. There have been similar findings for avian species (Johnson and Lanyon 1999). While our calculated rates are faster, it is not entirely surprising given that this is an intraspecific comparison, for which other empirical studies have found high rates (Lambert et al. 2002; Howell et al. 2003; Ho et al. 2005; Mao et al. 2006, 2007a, b). We suggest that it is not prudent to assume equal mutation rates across protein-coding mitochondrial genes for all species.

The Bayesian analysis produced a rate of divergence of 7.68–20.47% per my for*nd2*, more than double the other calculated estimates for the upper limit. It suggests that our use of the TrN model produced conservative estimates. However, 7.68–20.47% per my may not be an overestimate of the rate, as Bayesian analyses are often considered to be robust. There have been recent studies showing elevated rates of divergence are possible and likely because there may be an acceleration of the molecular clock on short time scales (Peterson and Masel 2009). Nonetheless, we have preferred to use the conservative rates in the interpretations.

## Conclusion

The data show that EH contain Blacknose Dace from two genetically distinct refugia. We conclude that*R. atratulus*recolonized EH during a single founding event from a single refugium. The original founding population spread into EH during the early stages of deglaciation via the temporary river connecting these three major drainage basins. And as the data suggest, a parallel situation of a single refugium recolonization was likely occurring in WH, but from a different refugium. This founding event was followed, approximately 9,000 years later, by a recolonization event introducing haplotype G to the Housatonic basin approximately 4,738–12,206 years BP. Further analysis of populations nearby Connecticut and New York drainage basins, for example, drainages in northern New Jersey and eastern Pennsylvania, will elucidate the location of their respective source of glacial refugium.

This study provides a baseline for a more extensive study of genetic diversity of fishes within New England. Now that we have concluded that the population of*R. atratulus*that recolonized Connecticut's drainage basins came from one refugium, we can begin to explore the possibilities of where this refugium might have been located. Our rate of mutation for*nd2*will further help this exploration. Knowledge of the process of freshwater fish recolonization of previously glaciated areas will fill gaps in our understanding of fish evolution, and our use of varve chronology to calculate divergence sets the stage for future work to create an accurate portrait of the postglacial recolonization of the northeast. This data suggest a very likely pathway for other freshwater fishes to have taken during the recolonization process, as the temporary glacial river connection to these three major drainage basins appear to have been the gateway to New England. We plan to continue to characterize the genetic diversity of Blacknose Dace throughout its modern range in order to draw further conclusions about the phylogeography of this species as a whole.

## Appendix A

**Table A1:** This table contains the information by sampling site for the Connecticut specimens

	Locations	Latitude	Longitude	*n*	No. of haplotypes	Drainage basin	Haplotype diversity (Hd)	Nucleotide diversity (π)	Average no. of bp differences within group
Total population	-	-	-	194	38	-	0.755 (0.032)	0.00158 (0.00016)	1.64
BR	Blackberry River	42.000819	–73.219711	8	6	Housatonic	0.893 (0.111)	0.00353 (0.00071)	3.68
DB	Deep Brook	41.392333	–73.332042	8	5	Housatonic	0.857 (0.108)	0.00237 (0.00081)	2.46
NRTa	Tributary to Naugatuck River	41.456153	–73.061944	10	2	Housatonic	0.200 (0.154)	0.00038 (0.00030)	0.40
PB	Pond Brook	41.458603	–73.326647	7	5	Housatonic	0.905 (0.103)	0.00430 (0.00069)	4.48
PgR	Pomperaug River	41.470939	–73.254992	7	4	Housatonic	0.810 (0.130)	0.00403 (0.00074)	4.19
PtR	Pootatuck River	41.406717	–73.272286	5	5	Housatonic	1.00 (0.126)	0.00384 (0.00099)	4.00
8ME	Eightmile River East Branch	41.442883	–72.305058	8	3	Connecticut	0.607 (0.164)	0.00065 (0.00021)	0.68
8MW	Eightmile River West Branch	41.441706	–72.332586	2	1	Connecticut	0	0	0
BB	Bunnel (Burlington) Brook	41.782447	–72.921856	5	1	Connecticut	0	0	0
BM	Beaver Meadow Brook	41.45795	–72.525672	2	1	Connecticut	0	0	0
CR	Coginchog River @ Creamry Road	41.443531	–72.687956	14	3	Connecticut	0.275 (0.148)	0.00064 (0.00035)	0.67
CR-M	Coginchog River @ Merriam Farm	41.540072	–72.684722	6	5	Connecticut	0.933 (0.122)	0.00282 (0.00073)	2.93
CR-W	Coginchog River @ Wadsworth	41.535731	–72.687042	6	3	Connecticut	0.733 (0.155)	0.00122 (0.00044)	1.27
MR	Mattabesset River	41.619325	–72.794481	16	2	Connecticut	0.233 (0.126)	0.00022 (0.00015)	0.23
SA	Salmon River	41.552794	–72.448908	16	5	Connecticut	0.808 (0.053)	0.00111 (0.00015)	1.16
SB	Salmon Brook	41.943817	–72.796069	9	3	Connecticut	0.417 (0.191)	0.00064 (0.00034)	0.67
SC	Scantic River	41.917022	–72.556003	6	1	Connecticut	0	0	0
StR	Still River	41.920125	–73.063658	10	3	Connecticut	0.378 (0.181)	0.00058 (0.00032)	0.60
HOP	Hop River trib-Bear Swamp Brook	41.741331	–72.341622	1	1	Thames	0	0	n/c
JB	Jordan Brook	41.987547	–72.017106	9	1	Thames	0	0	0
MB	Mason Brook	41.833236	–72.243056	12	2	Thames	0.545 (0.062)	0.00052 (0.00006)	0.54
MsR	Moosup River	41.718719	–71.898758	5	2	Thames	0.600 (0.175)	0.00058 (0.00017)	0.60
OB	Obwebtuck Brook	41.689561	–72.177931	9	2	Thames	0.222 (0.166)	0.00021 (0.00016)	0.22
WtB	Wheatons Brook	41.924281	–71.917205	8	3	Thames	0.607 (0.164)	0.00065 (0.00021)	0.68
YR	Yantic River	41.558594	–72.121397	5	3	Thames	0.700 (0.218)	0.00077 (0.00029)	0.80
*R. cataractae*	Coginchog River @ Merriam Farm	41.540072	–72.684722	6	1	Connecticut	0	0	0

**Table A2:** Summary of AMOVA statistics

**Table A3:** The number of individuals per site that represent each haplotype within Connecticut's*R. atratulus*for*ctr*. The top row indicates the river drainage basin with abbreviations for each of the sites given in the row below (full names in Table A1)

## References

[b1] Aldenhoven JT, Miller MA, Corneli PS, Shapiro MD (2010). Phylogeography of ninespine sticklebacks (*Pungitius pungitius*) in North America: glacial refugia and the origins of adaptive traits. Mol. Ecol.

[b2] Avise JC, Walker D (1998). Pleistocene phylogeographic effects on avian populations and the speciation process. Proc. R. Soc. Lond. B.

[b3] Avise JC (2000). Phylogeography: the history and formation of species.

[b4] Balco G, Schaefer JM (2006). Cosmogenic-nuclide and varve chronologies for the deglaciation of southern New England. Quat. Geochronol.

[b5] Balco G, Briner J, Finkel RC, Rayburn JA, Ridge JC, Schaefer JM (2009). Regional beryllium-10 production rate calibration for late-glacial northeastern North America. Quat. Geochronol.

[b6] Benner JS, Ridge JC, Taft NK (2008). Late pleistocene freshwater fish (cottidae) trackways from New England (USA) glacial lakes and a reinterpretation of the ichnogenus broomichnium kuhn. Palaeogeogr. Palaeoclimatol. Palaeoecol.

[b7] Benner JS, Ridge JC, Knecht RJ (2009). Timing of post-glacial reinhabitation and ecological development of two New England, USA, drainages based on trace fossil evidence. Palaeogeogr. Palaeoclimatol. Palaeoecol.

[b8] Boothroyd J, Freedman JH, Brenner HB, Stone JR, Murray DP (1998). The glacial geology of southern Rhode Island. Guidebook to field trips in Rhode Island and adjacent regions of Connecticut and Massachusetts.

[b9] Clement M, Posada D, Crandall K (2000). TCS: a computer program to estimate gene geneaolgies. Moleculare Ecology.

[b10] Dawson AG (1992). Ice Age Earth: Late Quarternary Geology and Climate, (1-293).

[b11] Drummond AJ, Rambaut A (2007). BEAST: Bayesian evolutionary analysis by sampling trees. Bmc Evol. Biol.

[b12] Excoffier L, Lischer HEL (2010). Arlequin suite ver 3.5: a new series of programs to perform population genetics analyses under linux and windows. Mol. Ecol. Res.

[b13] Fu YX (1997). Statistical tests of neutrality of mutations against population growth, hitchhiking and background selection. Genetics.

[b14] Gaudeul M (2006). Disjunct distribution of hypericum nummularium l. (hypericaceae): molecular data suggest bidirectional colonization from a single refugium rather than survival in distinct refugia. Biol. J. Linnean Soc.

[b15] Gilhen J, Hebda A (2002). Distribution of Blacknose Dace,*Rhinichthys atratulus*, in nova scotia. Can. Field Nat.

[b16] Girard P, Angers B (2006). The impact of postglacial marine invasions on the genetic diversity of an obligate freshwater fish, the Longnose Dace (*Rhinichthys cataractae*), on the Quebec peninsula. Can. J. Fish. Aquat. Sci.

[b17] Gugerli F, Ruegg M, Vendramin GG (2009). Gradual decline in genetic diversity in swiss stone pine populations (*Pinus cembra)*across Switzerland suggests postglacial re-colonization into the alps from a common eastern glacial refugium. Bot. Helv.

[b18] Hewitt G (2000). The genetic legacy of the quaternary ice ages. Nature.

[b19] Ho SYW, Shapiro B, Phillips MJ, Cooper A, Drummond AJ (2007b). Evidence for time dependency of molecular rate estimates. Syst. Biol.

[b20] Ho SYW, Phillips MJ, Cooper A, Drummond AJ (2005). Time dependency of molecular rate estimates and systematic overestimation of recent divergence times. Mol. Biol. Evol.

[b21] Ho SYW, Heupink TH, Rambaut A, Shapiro B (2007a). Bayesian estimation of sequence damage in ancient DNA. Mol. Biol. Evol.

[b22] Ho SYW (2007). Calibrating molecular estimates of substitution rates and divergence times in birds. J. Avian Biol.

[b23] Hoarau G, Coyer JA, Veldsink JH, Stam WT, Olsen JL (2007). Glacial refugia and recolonization pathways in the brown seaweed fucus serratus. Mol. Ecol.

[b24] Howell N, Smejkal CB, Mackey DA, Chinnery PF, Turnbull DM, Herrnstadt C (2003). The pedigree rate of sequence divergence in the human mitochondrial genome: there is a difference between phylogenetic and pedigree rates. Am. J. Hum. Genet.

[b25] Hurt C, Anker A, Knowlton N (2009). A multilocus test of simultaneous divergence across the isthmus of panama using snapping shrimp in the genus alpheus. Evolution.

[b26] Johnson KP, Lanyon SM (1999). Molecular systematics of the grackles and allies, and the effect of additional sequence (cyt b and nd2). Auk.

[b27] Knecht RJ, Benner JS, Rogers C, Ridge JC (2009). *Surculichnus bifurcauda*n. Igen., n. Isp., a trace fossil from late pleistocene glaciolacustrine varves of the Connecticut river valley, USA, attributed to notostracan crustaceans based on neoichnological experimentation. Palaeogeogr. Palaeoclimatol. Palaeoecol.

[b28] Lambert DM, Ritchie PA, Millar CD, Holland B, Drummond AJ, Baroni C (2002). Rates of evolution in ancient DNA from adelie penguins. Science.

[b29] Lee WJ, Conroy J, Howell WH, Kocher TD (1995). Structure and evolution of teleost mitochondrial control regions. J. Mol. Evol.

[b30] Librado P, Rozas J (2009). Dnasp v5: a software for comprehensive analysis of DNA polymorphism data. Bioinformatics.

[b31] Mao L, Zabel C, Nebrich G, Wacker MA, Sagi D, Schrade P, Bachman S, Kowald A, Klose J (2006). Estimation of the mtdna mutation rate in aging mice by proteome analysis and mathematical modeling. Exp. Gerontol.

[b32] Meyer A, Morrissey JM, Schartl M (1994). Recurrent origin of a sexually selected trait in xiphophorus fishes inferred from a molecular phylogeny. Nature.

[b33] Miura O, Torchin ME, Bermingham E (2010). Molecular phylogenetics reveals differential divergence of coastal snails separated by the isthmus of panama. Mol. Phylogenet. Evol.

[b34] Moncrief ND, Lack JB, Van Den Bussche RA (2010). Eastern fox squirrel (*Sciurus niger*) lacks phylogeographic structure: Recent range expansion and phenotypic differentiation. J. Mammal.

[b35] Nei M, Kumar S (2000). Molecular evolution and phylogenetics.

[b36] Nelson JS, Crossman EJ, Espinosa-Perez H, Findley LT, Gilbert CR, Lea RN, Williams JD (2004). Common and scientific names of fishes from the United States, Canada, and Mexico.

[b37] Paxinos EE, James HF, Olson SL, Sorenson MD, Jackson J, Fleischer RC (2002). Mtdna from fossils reveals a radiation of Hawaiian geese recently derived from the Canada goose (*Branta canadensis*). Proc. Natl. Acad. Sci. U. S. A.

[b38] Peteet DM, Daniels RA, Heusser LE, Vogel JS, Southon JR, Nelson DE (1993). Late-glacial pollen, macrofossils and fish remains in northeastern USA – the younger dryas oscillation. Quaternary Sci. Rev.

[b39] Peters JL, Gretes W, Omland KE (2005). Late pleistocene divergence between eastern and western populations of wood ducks (*Aix sponsa*) inferred by the ‘isolation with migration’ coalescent method. Mol. Ecol.

[b40] Peterson GI, Masel J (2009). Quantitative prediction of molecular clock and k-a/k-s at short timescales. Mol. Biol. Evol.

[b41] Pfrender ME, Hicks J, Lynch M (2004). Biogeographic patterns and current distribution of molecular-genetic variation among populations of speckled dace,*Rhinichthys osculus*(girard). Mol. Phylogenet. Evol.

[b42] Posada D, Buckley TR (2004). Model selection and model averaging in phylogenetics: advantages of akaike information criterion and bayesian approaches over likelihood ratio tests. Syst. Biol.

[b43] Posada D (2008). Jmodeltest: phylogenetic model averaging. Mol. Biol. Evol.

[b44] Ridge JC, Ehlers J, Gibbard PL (2004). The quaternary glaciation of western new england with correlations to surrounding areas. Quarternary glaciations – extent and chronology, Part II North America.

[b45] Rowe KC, Heske EJ, Brown PW, Paige KN (2004). Surviving the ice: northern refugia and postglacial colonization. Proc. Natl. Acad. Sci. U. S. A.

[b46] Schmidt TR, Bielawski JP, Gold JR (1998). Molecular phylogenetics and evolution of the cytochrome b gene in the cyprinid genus lythrurus (actinopterygii: Cypriniformes). Copeia.

[b47] Smith GR, Dowling TE (2008). Correlating hydrographic events and divergence times of speckled dace (Rhinichthys: Teleostei: Cyprinidae) in the Colorado river drainage. Late Cenozoic Drainage History of the Southwestern Great Basin and Lower Colorado River Region: Geologic and Biotic Perspectives.

[b48] Soltis DE, Morris AB, Mclachlan JS, Manos PS, Soltis PS (2006). Comparative phylogeography of unglaciated eastern North America. Mol. Ecol.

[b49] Steele CA, Storfer A (2006). Coalescent-based hypothesis testing supports multiple pleistocene refugia in the pacific northwest for the pacific giant salamander (*Dicamptodon tenebrosus*). Mol. Ecol.

[b50] Stone BD, Borns H, Sibrabva V, Bowen DQ, Richmond GM (1986). Pleistocene glacial and interglacial stratigraphy of New England, long Island, and adjacent Georges Bank and Gulf of Maine. Quaternary Glaciations in the Northern Hemisphere, (Quarternary Science Reviews, 5, p.39-52).

[b51] Stone J, Stone BD, Lewis RS, Tracey RJ (1985). Late quaternary deposits of the southern quinnipiac-farmington lowland and Long Island Sound basin: their place in a regional stratigraphic framework, trip c. New England Intercollegiate Geological Conference 77th annual meeting.

[b52] Stone J, Digiacomo-Cohen M, Lewis RS, Goldsmith R, Murray DP (1998). Recessional moraines and the associated deglacial record of southeastern Connecticut. Guidebook to field trips in Rhode Island and adjacent regions of Connecticut and Massachusetts.

[b53] Stone JR, Schafer JP, London EH, Digiacomo-Cohen ML, Lewis RSAT, Woodrow B (2005). Quaternary geologic map of Connecticut and Long Island Sound basin. United States geological survey.

[b54] Tamura K, Nei M (1993). Estimation of the number of nucleotide substitutions in the control region of mitochondrial-DNA in humans and chimpanzees. Mol. Biol. Evol.

[b55] Tamura K, Dudley J, Nei M, Kumar S (2007). Mega4: molecular evolutionary genetics analysis (mega) software version 4.0. Mol. Biol. Evol.

[b56] Tang LZ, Wang LY, Cai ZY, Zhang TZ, Ci HX, Lin GH, Su JP, Liu JQ (2010). Allopatric divergence and phylogeographic structure of the plateau zokor (*Eospalax baileyi*), a fossorial rodent endemic to the qinghai-tibetan plateau. J. Biogeogr.

[b57] Templeton AR (1998). Nested clade analyses of phylogeographic data: testing hypotheses about gene flow and population history. Mol. Ecol.

[b58] Venkatesan M, Westbrook CJ, Hauer MC, Rasgon JL (2007). Evidence for a population expansion in the west nile virus vector culex tarsalis. Mol. Biol. Evol.

[b59] Wright S (1943). Isolation by distance. Genetics.

